# The Genesis and Treatment of Hyperpyrexia

**Published:** 1894-11-24

**Authors:** 


The Hospital

An Institutional Journal of

The Medical Sciences and Hospital Administration, with

AN EXTRA NURSING SUPPLEMENT.

Vol. XVII.—No. 426.] [Nov. 24, 1894.

### The Genesis and Treatment of Hyperpyrexia.

The causes of pyrexia, that is, of abnormal elevation of temperature in the human organism are believed to be pretty generally understood, and the treatment of the condition is fairly uniform throughout the profession, at any rate as to its general principles. It may be doubted, however, whether our knowledge is so full and assured as we suppose it to be, and as it is eminently desirable that it should be. Be that as it may in the case of ordinary pyrexia, it is certain that hyperpyrexia, elevation of temperature running up to 107 deg., 108 deg., 109 deg., and even considerably higher than these figures, is a phenomenon which often presents itself to the eye without at the same time suggesting to the mind a clear picture of its causation.

Dr. Hale White thinks he has thrown some light upon this obscure, but deeply interesting series of problems, and readers will probably be inclined to agree with him. The nervous system, he tells us, is the part of the organism the interrogation of which has produced answers and explanations which seem to be both scientific and practical. The corpora striata are affirmed to be thermogenetic centres; and in this fundamental fact is found the key to unlock the mysteries of "paradoxical" and formerly inexplicable vagaries of temperature. Thermotaxic, distinguished from thermogenetic, centres are also as believed by some experimenters to be discoverable in the cerebral cortex. That position is not, however, proved as yet. But that the corpora striata are centres which control the generation of heat in the muscles and elsewhere, and that those portions of the brain which are organically continuous with them, the crura, the pons, and the spinal cord, are intimately associated with this thermogenetic function is practically established beyond a doubt. The evidence for this contention advanced by Dr. Hale White is both experimental and clinical. What is surmised and demonstrated by experiment in the physiological laboratory is fully confirmed at the bedside.

In twenty-seven experiments made by Dr. Hale White, involving damage to one or other of the corpora striata, there was a marked rise of general temperature in every instance, while in seven of the cases the thermometer indicated temperatures of 106 deg. and upwards. These results have been confirmed by Reichert and others. In like manner it has been experimentally demonstrated that lesions of the crura cerebri, of the pons and of the upper part of the spinal cord, as well as of the cerebral cortex, have resulted in hyperpyrexial conditions strictly comparable in degree to the abnormally high temperatures sometimes noted as of cerebral origin at the bedside. Some of these experimentally produced temperatures, notably those recorded by Dr. J. H. Bryant, have reached 107 deg., 108 deg., and even 109 deg. Fahr. If now we turn for a moment to the records of clinical experience we are reminded that in meningitis a temperature of 108 deg. is not very uncommon, in epilepsy 109 deg. has been reached, whilst in hysteria, chorea, and delirium tremens as high and even higher temperatures than these have been recorded by various observers. The general contention here maintained is that temperatures which are really hyperpyrexial and not merely pyrexial, are for practical purposes always of nervous origin, and must be treated accordingly.

In these demonstrated facts there are, it seems to us, the clearest possible indications for treatment, and for treatment of the most definite kind. In all cases of positive hyperpyrexia the temperature must be lowered by the most certain method at command. What is that method? It is the use of water in the form of the bath. The cold bath, which in many instances is to be preferred to affusion or to the graduated bath, is to be employed decisively and without fear. Very striking are some of the facts recorded by Dr. Hale White in this connection. For example, Dr. J. H. Bryant treated fifty-six cases of rheumatic hyperpyrexia by means of the cold bath, and of these as many as thirty-three recovered. On the other hand, seven cases of the same class were treated without the cold bath, and every one of them died.

On the question of drugging in hyperpyrexial states Dr. Hale White is equally decided. Not only does he affirm that "no dependence" whatever is to be placed on antipyretic drugs, but he goes further. Those drugs are, he says, in large quantities, "direct cardiac poisons," and some of them, moreover, have the fatal property of being destructive to the red blood corpuscles. Phenacetine, acetanilid, phenazone, and certain other popular and strictly modern antipyretics should, he considers, never be used in cases of true hyperpyrexia, and very seldom even in the ordinary specific fevers. Nay more, Dr. Hale White firmly urges the now somewhat familiar but still quite recent suggestion that the moderate pyrexia of ordinary fevers, such as typhoid and others, may be of real service to the patient, and powerful drugs administered for the forcible lowering of the temperature may be productive of much more harm than good. We confess that this contention appeals strongly to the mind imbued with the principles of modern physiology. In these fevers, and in most conditions of moderate pyrexia, it is quite easy to understand how cold affusion, or in severe cases the cold bath, may be of infinite service, both by lowering temperature and toning up the nervous system. Very different, however, may be the consequences of administering drugs internally which check oxidation and originate physiological processes of which we know neither the rationale, nor the beginning nor the end.

It is not to be supposed that the members of the British Medical Association, to whom Dr. Hale White's paper was addressed, entirely coincided with all his very modern contentions; nor was it desirable that they should. The old joined issue with the new in the debate; and good grounds were affirmed for continuing the use of quinine, the salicylates, and other compounds of which experience has established the value beyond a doubt. It is certain, however, that the routine treatment of pyrexia as well as of hyperpyrexia is doomed, and that more than ever must scientific practitioners take their cue from the physiological laboratory.

